# Physical-Mechanical Properties of Bamboo Fiber Composites Using Filament Winding

**DOI:** 10.3390/polym13172913

**Published:** 2021-08-29

**Authors:** Wenfu Zhang, Cuicui Wang, Shaohua Gu, Haixia Yu, Haitao Cheng, Ge Wang

**Affiliations:** 1 Zhejiang Forestry Research Institute, Hangzhou 310023, China; zhangwenfu542697@163.com (W.Z.); ivyyhx@126.com (H.Y.); 2 International Centre for Bamboo and Rattan, Beijing 100102, China; cuicui124@163.com (C.W.); htcheng@icbr.ac.cn (H.C.); wangge@icbr.ac.cn (G.W.)

**Keywords:** twisting bamboo fiber (TBF), filament winding, NOL ring, epoxy composite

## Abstract

In order to study the performance of the bamboo fiber composites prepared by filament winding, composites reinforced with jute fiber and glass fiber were used as control samples. The structure and mechanical properties of the composites were investigated by scanning electric microscope (SEM), tensile testing, bending testing, and dynamic mechanical analysis. The results demonstrated that the bamboo fiber composites exhibited lower density (0.974 g/cm^3^) and mechanical properties in comparison of to fiber composite and glass fiber composite, because the inner tissue structure of bamboo fiber was preserved without resin adsorbed into the cell cavity of fibrous parenchyma. The bamboo fibers in composites were pulled out, while the fibers in the surface of composites were torn, resulting in the lowest mechanical performance of bamboo fiber composites. The glass transition temperature of twisting bamboo fiber Naval Ordnance Laboratory (TBF-NOL) composite (165.89 °C) was the highest in general, which indicated that the TBF circumferential composite had the best plasticizing properties and better elasticity, the reason being that the fiber-reinforced epoxy circumferential composite interface joint is a physical connection, which restricts the movement of the molecular chain of the epoxy matrix, making the composite have a higher storage modulus (6000 MPa). In addition, The TBF-NOL had the least frequency dependence, and the circumferential composite prepared by TBF had the least performance variability. Therefore, the surface and internal structures of the bamboo fiber should be further processed and improved by decreasing the twisting bamboo fiber (TBF) diameter and increasing the specific surface area of the TBF and joint surface between fibers and resin, to improve the comprehensive properties of bamboo fiber composites.

## 1. Introduction

Natural fibers (NFs) are becoming widely used due to their low density, excellent mechanical properties, low price, durability, sustainability, and biodegradability [1]. Especially, by replacing the use of glass fiber in composite, NFs can play a positive role in energy saving and emission reduction, which will allow for green processing and recycling in the composite industry which is driving the increase in NFs’ use in various applications such as furniture, architecture, automobiles, etc. [2,3,4].

The bamboo fibers isolated and prepared from bamboo have good mechanical properties. The tensile strength and tensile modulus of single bamboo fiber can be more than 1.43–1.69 GPa and 32–34.6 GPa, respectively, and the elongation at break of single bamboo fiber was 4.3–9.7%. Based on these properties, bamboo fiber is regarded as natural glass fibers [5,6,7]. Due to the unique properties and excellent mechanical performance, bamboo fiber has attracted significant attention in the fiber reinforced composite industry [8,9,10]. Many studies have been carried out regarding aspects of morphology and size of the bamboo fiber, surface of the bamboo fiber and its interface modifications with plastic resins, types and amounts of matrices, and the composite molding process and performance [11,12]. Specifically, bamboo fiber/epoxy composites prepared with epoxy as the matrix have multiple advantages, such as being light-weight, high strength, good fatigue resistance, good buffer performance, low cost, low energy consumption, and non-toxicity. In many applications, they can replace glass fiber composites, exhibiting advantages of regeneration and recycling of bamboo fiber and can be applied in automotive substructures, electrical shells, decoration, and packaging materials [13,14].

As the economy develops, the demand for multiple types of bamboo fiber composites is increasing, and the quality requirements are becoming more stringent. However, due to the inherent defects of bamboo and processing technology, bamboo fiber composites have many distinct drawbacks, such as insufficient product design, poor performance stability, and poor applicability in various markets [15,16]. In particular, as a typical plant fiber, bamboo fiber shows strong hygroscopicity, causing a certain gap between the application performance of bamboo fiber and other fiber composites [17,18]. Therefore, it is necessary to explore new technology for the manufacturing of bamboo fiber composites to develop stable bamboo fiber composites.

Fiber filament winding is a key technology in the fabrication of advanced composites through winding fibers onto the modules to prepare the composites. This technology can be applied to prepare heterotypic composites with excellent performance, stable structure, and special application, realizing the manufacture of composites without external force [19,20]. To evaluate the fiber properties and the interface between the fiber and resin to optimize the curing process, a common method was adopted, which included the preparation of the hoop winding Naval Ordnance Laboratory (NOL, for short) ring and analysis of the product properties to provide basic process parameters for winding composites, while directly reflecting the structure and performance characteristics of filament winding composites. In this study, bamboo fiber was first processed into twisting bamboo fiber (TBF, for short) and then wound into the NOL ring, which was comparatively analyzed with the glass fiber and jute fiber to provide reference and support for the application of filament winding of bamboo fibers.

## 2. Materials and Methods

### 2.1. Materials

The materials used in this study include laboratory-made twisted bamboo fiber, jute fiber (commercially purchased), and glass fibers (commercially purchased). The glass fibers were alkali-free assembled rovings with a roving density of 1080 tex.

### 2.2. Composite Preparation NOL

**Figure 1:**
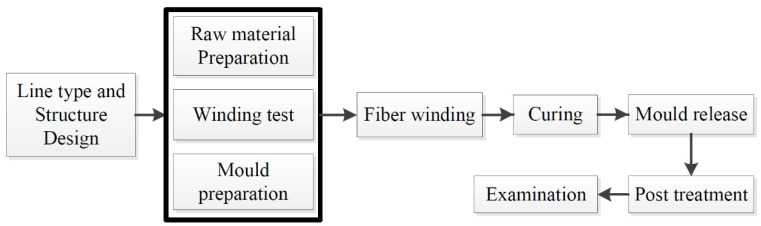
NOL ring fabrication preparation.

The fabrication process of the fiber-reinforced epoxy composites, circumferential filament winding composite, is shown in Figure 1.

**Figure 2:**
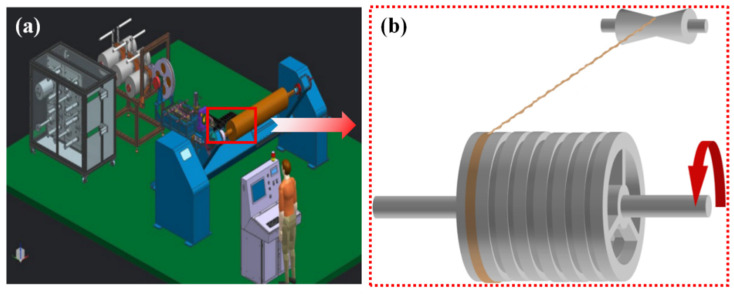
NOL ring preparation: (**a**) winding process schematic; (**b**) NOL ring winding.

The process is as follows. Compile parameters such as winding pattern and winding speed of the equipment. Prepare the resin with epoxy YD-127 and curing agent EC-201 in a proportion of 4:1, stir evenly, pour into the winding machine glue tank, and adjust the sizes of the glue-dipping and glue-scraping apertures to reduce its influence on the fiber. Load the dry continuous fiber on the mechanical tension yarn rack, adjust the mechanical tension controller, and wrap the NOL ring according to the designed winding scheme (Figure 2). During the winding process, remove the excess glue on the surface of the NOL ring with the rubber scraper. After completing the fiber winding, shear the fiber and stop the winding machine, but keep the rotation of the winding shaft. Install the on-line curing unit, keeping the surface temperature of the NOL ring at 120 °C for 30 min while on-line curing, then reduce to room temperature, remove the NOL ring samples, and carry out post-processing or further ripening; the fiber weight content of TBF-NOL, JF-NOL, and GF-NOL were 55%, 57%, and 60%, respectively. After complete curing of the resin, let it stand for at least 7 days, and then carry out the mechanical performance test.

### 2.3. Mechanical Performance Testing

**Figure 3:**
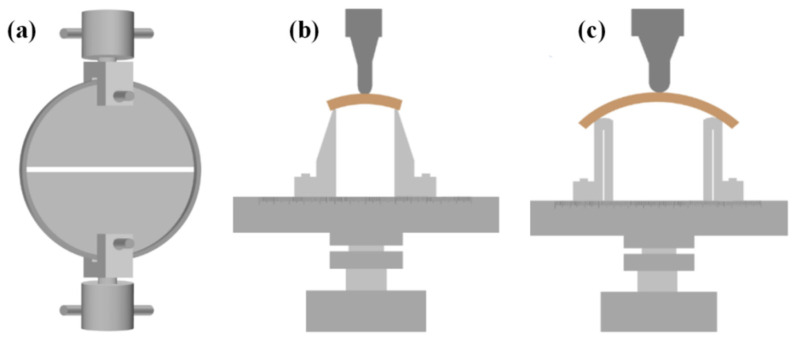
Testing of mechanical performance of NOL rings. (**a**) Tensile performance testing; (**b**) shear performance testing; (**c**) bending performance testing.

The tensile performance of NOL rings was tested according to ASTM D2290-19 with the mechanical testing machine (Instron 5582, Instron instruments, Boston, MA, USA) using a 100 kN sensor and tensile plate fixture. In the process, each testing group contained 6 samples and the loading speed was adjusted to ensure sample failure was completed in 60–90 s. The shear and bending performances of the NOL rings were tested based on the references of ASTM D2344/D2344M-16 and ISO 14125 with the Instron 5848 mechanical testing machine using a 2 kN sensor and three point bending fixture. For the shear and bending performance tests, the span was four times and sixteen times the thickness of the sample, respectively, and the diameter of the loading and supporting fixture was 4 mm and 10 mm, respectively. In the testing process, each group contained 12 samples, which were placed on the support with the convex surface upward, and the loading speed was adjusted to ensure sample failure was completed in 60–90 s. The testing process is shown in Figure 3.

### 2.4. Dynamic Mechanical Analysis (DMA)

The interface performance of the composite was analyzed by thermal dynamic mechanical analysis. The storage modulus (*E*′), loss modulus (*E*″), and loss factor (tan *δ*) of composite filaments were carried out according to ASTM D 7028-07^ε1^ using a dynamic mechanical analyzer(DMA Q800, TA instruments Inc, New Castle, DL, USA). The three-point bending fixture was applied in the analysis because it did not produce the holding effect and was suitable for harder samples and characterizing the tensile and compression loss characteristics of the composites with laminated structures. Specific testing parameters included the three-point bending loading mode, where the temperature increased from room temperature to 240 °C, the heating rate was 3 °C/min, the amplitude was 15 μm, and the frequencies were 1 Hz, 4 Hz, 7 Hz, and 10 Hz, respectively. The sample size was 60 × 4 × 3 mm, and each sample from each group was repeated three times.

### 2.5. Morphology Observation

The instrument used to analyze the surface morphology of the composite filaments and the interfacial quality between phases was a field emission environmental scanning electron microscope (ESEM; XL30 ESEM-FEG; FEI Co., Hillsboro, OR, USA). The samples were sputter coated with a thin layer of gold in a vacuum chamber for conductivity before examination and were analyzed in a vacuum chamber that was less than 5 × 10^−5^ Pa at a voltage of 10 kV. 

### 2.6. Statistical Analysis

The findings obtained (strength tests, water contact angle, color changes) were subjected to statistical treatment in the origin 2018. The results were presented as average values ± standard deviation (SD). 

## 3. Results and Discussion

### 3.1. Mechanical Performance

The physical mechanical performance of the NOL rings prepared from three fiber materials were tested, and the results are shown in Table 1. According to Table 1, the density of the TBF-NOL ring is slightly lower than that of the JF-NOL ring, and the density of the GF-NOL ring is 1.95 times higher than that of the TBF-NOL ring with the same winding layers and resin content. The mechanical performance of the tensile strength, shear strength, and bending strength was analyzed, and GF-NOL had the highest mechanical properties compared with other composites, which were 19.42, 2.05, and 7.86 times that of the TBF-NOL composites, respectively. The strength of the TBF-NOL ring is lower than the GF-NOL and JF-NOL rings. The surface bending strain is the lowest, indicating higher rigidity, which is better than that of the JF-NOL GF-NOL rings.

**Figure 4:**
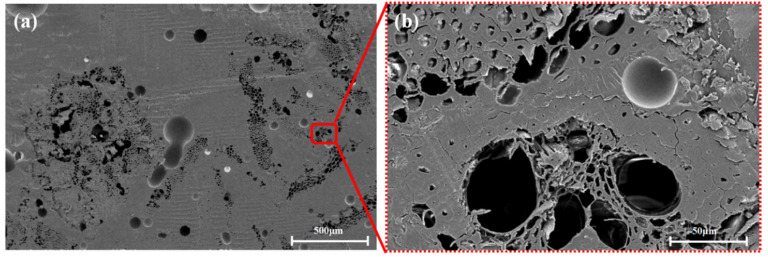
Microscopic morphology of cross-section of TBF-NOL: (**a**), 50×; (**b**), 500×.

**Figure 5:**
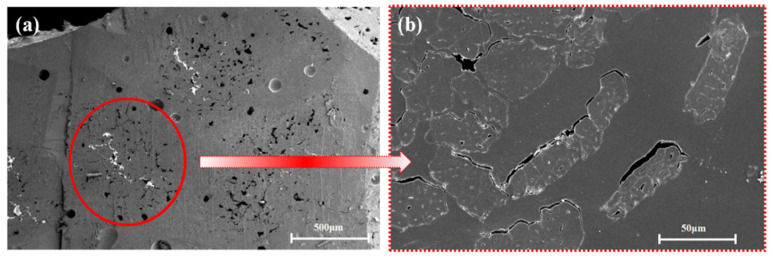
Microscopic morphology of cross-section of JF-NOL: (**a**), 50×; (**b**), 500×.

**Figure 6:**
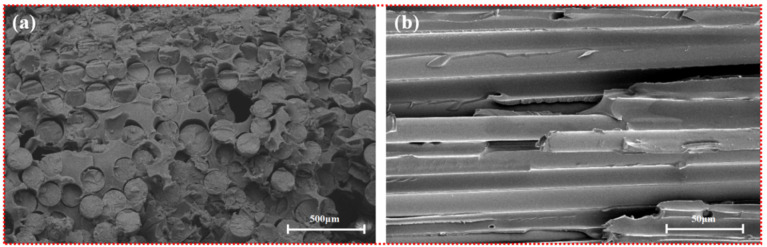
Microscopic morphology of GF-NOL: (**a**), 500×; (**b**), 500×.

As shown in Figure 4, Figure 5 and Figure 6, the fibers of the NOL ring prepared from TBF, JF, and GF were mixed well with epoxy. Inside the TBF-NOL and JF-NOL rings existed a small amount of resin which was primarily wrapped fiber bundles. Additionally, bamboo fiber has its own parenchyma and conduit structure and resin failed to immerse into the internal pores of the bamboo fiber, while jute fiber also had small pores, smaller than those of bamboo fiber. The epoxy distributed around each fiber in the cross-section of the GF-NOL ring, and the fibers were well dispersed in the resin, forming good bonding properties.

**Figure 7:**
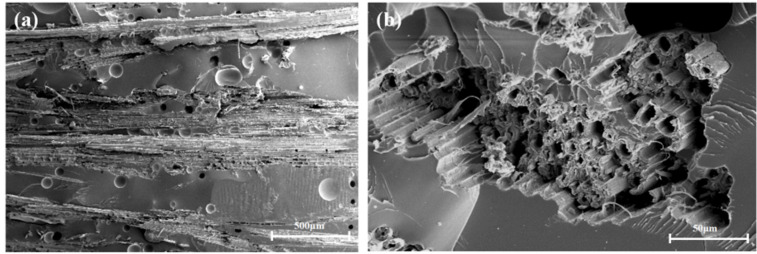
Morphology of the TBF-NOL circumferential composite: (**a**), 50×; (**b**), 500×.

Figure 7 shows the microscopic morphology of TBF-NOL. As shown in Figure 6a of the cross-section of bamboo fiber, the inner tissue structure was preserved in the bamboo fiber with no resin entering into the cell cavity of the parenchyma, and the resin in the two sides of the fiber cured into the pores with a diameter of about 100 μm. It can be observed that a fiber was capsulated by the polymer matrix, fibers were pulled out, surface fibers were torn, and inner fibers were extracted, which was also stated in Reference [21]. Therefore, other than only tensile force in the TBF preparation, other factors should also be taken into consideration, including reducing the diameter, improving the specific surface area of TBF, and increasing the surface interaction between fiber and resin.

### 3.2. Thermodynamic Mechanical Performance

Analysis of the frequency dependence of the storage modulus. The *k* value was calculated according to Equation (1) as the correlation coefficient of the frequency dependence of the storage modulus.

(1) $$E^{\prime }=klgf+b$$

Analysis of the relationship between glass transition temperature and frequency. The apparent activation energy was calculated according to Equation (2) obtained by transforming the Arrhenius formula and was used to characterize the relationship between glass transition temperature and frequency.

(2) $$E_{a}=-R\cdot \frac{d\left(\ln f\right)}{d\left(1/T_{g}\right)}$$

where *Ea* is the glass transition apparent activation energy (kJ/mol), *R* is the universal gas constant, (8.314 × 10^−3^ kJ/(mol·K), *f* is the frequency (Hz), and *Tg* is the glass transition temperature of the fiber composite (K) at frequency *f.*

**Figure 8:**
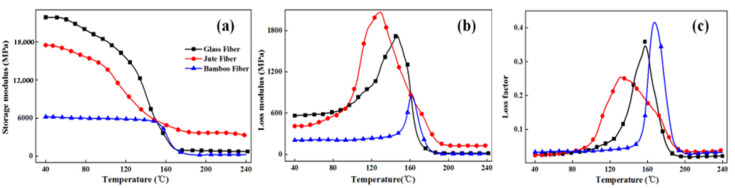
Thermodynamic mechanical performances of circumferential composites at single frequency: (**a**), Storage modulus; (**b**), Loss modulus; (**c**), Loss factor.

The dynamic mechanical performance test results of three fiber circumferential composites at 1 Hz frequency are shown in Figure 8. As shown in Figure 8a, among the NOL rings prepared from three fibers, the E′ value of TBF-NOL ring is the lowest (6187 MPa), while that of GF-NOL ring is the highest (21,857 MPa), which was 3.53 times higher than that of the TBF circumferential composite. The fiber-reinforced epoxy circumferential composite interface was physically connected, which inhibited the movement of epoxy matrix molecular chains, enabling a higher storage module of the composite.

In addition, the storage modulus E′ of NOL rings prepared from three fibers decreased with the increase in temperature, but when the temperature increased from 40 to 150 °C, the storage moduli of TBF-NOL, JF-NOL, and GF-NOL decreased by 12.88%, 67.13%, and 74.26%, respectively. The storage modulus of the TBF circumferential composite slightly decreased, while the storage moduli of the GF and JF circumferential composite showed obvious decreases. This may be because the interface between the epoxy resin and fiber was significantly affected by heat. The interface between fiber and resin in the TBF composite was good, which indicated that the thermal stability of the TBF composites composite was better at low and medium temperatures compared with high temperature. The storage modulus of the three materials were similar at around 150 °C. When the temperature exceeded 150 °C, the storage modulus of the JF composite was the highest. As the temperature increased, the storage modulus of the three materials continued to decrease. When the temperature increased from 40 °C to 170 °C, the storage modulus of the TBF circumferential composite decreased rapidly, especially between 150 °C and 170 °C. At 240 °C, the storage modulus of TBF, JF, and GF composites decreased by 6.36%, 81.49%, and 96.69%, respectively.

As shown in Figure 8b, the loss modulus of GF and JF circumferential composites were higher than that of TBF circumferential composites in the temperature range of 40–160 °C, which indicated that the interfaces of GF epoxy and JF epoxy were superior to the TBF epoxy interface. In addition, when the molecular chains exhibited more frictional movement, it created more thermal energy of molecular motion, reflecting a larger loss modulus. According to Figure 7b, the loss peak areas of the three circumferential composites (TBF-NOL, JF-NOL, and GF-NOL) areas were 39,800.62, 145,779.01, and 116,596.58 (MPa·min), respectively. The loss area of TBF-NOL was the smallest, which indicated that the interface bonding was the weakest.

As shown in Figure 8c, the glass transition temperatures of TBF-NOL, JF-NOL, and GF-NOL were 165.89 °C, 130.22 °C, and 157.27 °C, respectively. The loss factor of TBF-NOL was the lowest in general, which indicated that the TBF composite had better elasticity and larger TBF diameter, which made the loss factor of the TBF composite lower. However, the glass transition temperature of the TBF circumferential composite was the highest, which indicated that the TBF circumferential composite had the best plasticizing properties and better elasticity.

**Figure 9:**
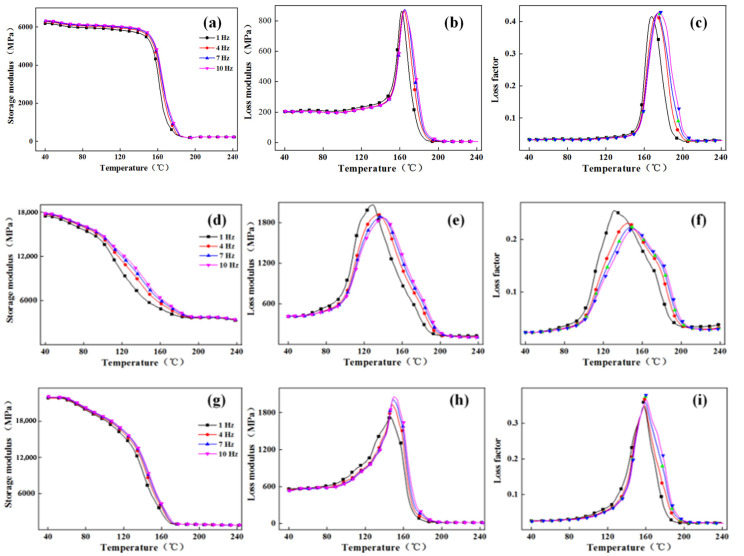
DMA performance data at various frequencies: (**a**–**c**) TBF-NOL composite; (**d**–**f**) JF-NOL composite; (**g**–**i**) GF-NOL composite.

Figure 9 illustrated DMA curves of TBF-NOL, JF-NOL, and GF-NOL at different frequencies (from left to right: storage modulus, loss modulus, loss factor). As observed, frequency showed the same effect on the three circular composites. With the increase in frequency, the peak of storage modulus, loss modulus, and loss factor gradually moved to high temperatures. This was because when the composite was under the external load, the inner molecular chains and interface acted together to resist the external force. With higher frequency, the action period was shorter, and internal molecular chain recombination was faster, leading to smaller composite changes and a larger storage modulus.

The correlation coefficient k between the storage modulus and frequency is calculated according to Equation (1). Temperatures of 40 °C and 120 °C were selected as the glassy-state temperature, and 200 °C was selected as the rubbery-state temperature (Table 2). The *k* value of the TBF composite was the lowest at different temperatures, while the *k* value was the highest, indicating the least frequency dependence of the storage modulus of the TBF-NOL composite and the least performance variability of the circumferential composite prepared from TBF.

**Figure 10:**
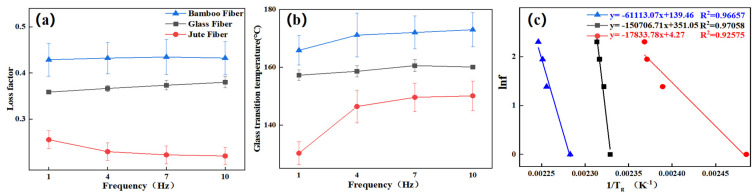
Loss factors and glass transition temperatures of circumferential composites at different frequencies: (**a**) loss factor vs. frequency; (**b**) Tg vs. frequency; (**c**) apparent activation energies of circumferential composites.

Figure 10 shows the relationship between frequency and loss factor, frequency, and glass transition temperature of the composites, respectively. With the frequency increased, the change trends of loss modulus and loss factor of the three fiber-refined composites were different, in which the peak values of the loss modulus and loss factor of the TBF circumferential composite increased with frequency, and the temperature corresponding to the peak value slightly increased. The loss modulus peak value of the JF composite decreased slightly with the increase in frequency, and the loss factor peak value gradually increased with the increase in frequency.

With the increase in frequency, the observation time of the internal molecular motion of the three circumferential composites lagged behind the relaxation time of the molecular chain sliding motion. To reduce or eliminate the influence of hysteresis, it was necessary to increase the temperature to ensure relative free motion of the molecular chain. At the temperature of 160 °C, the composite performance changed rapidly. With the increase in frequency, the reaction time of the polymer segment motion of composite was less than the observation time, which resulted in a change in loss modulus and loss factor of the TBF circumferential composite in the glass transition stage, which was also stated in Reference [22].

Figure 10c shows the activation energies of the circumferential composites calculated by Equation (2). The activation energies of TBF, JF, and GF circumferential composites were 508.12 kJ/mol, 148.28 kJ/mol, and 1253.04 kJ/mol, respectively. It is known that lower activation energies correlate with better interfacial bind properties, and thus, it can be concluded that the interface of the TBF and JF circumferential composite was superior to that of the GF circumferential composite. Therefore, the interface between plant fiber and epoxy is better than that between glass fiber and epoxy. Additionally, the interface between the fibers and resin of the JF circumferential composite exhibited the best performance due to few internal pore defects. Therefore, the surface and internal structure of the bamboo fiber should be further processed and improved to improve the mechanical properties of the bamboo fiber composite.

## 4. Conclusions

In this study, TBF-NOL, JF-NOL, and GF-NOL ring composites were successfully manufactured by filament winding. In the comparative analysis of the structures, bamboo fiber has its own parenchyma and conduit structure and resin failed to immerse into the internal pores of the bamboo fiber, while jute fiber also had small pores, smaller than those of bamboo fiber. The epoxy distributed around each fiber in the cross-section of the GF-NOL ring, and the fibers were well dispersed in the resin, forming good bonding properties.

The tensile strength, shear strength, and bending strength of TBF-NOL was 5.15%, 48.78%, and 12.72% of GF-NOL, respectively. It can be concluded by activation energy that the interface of the TBF and JF circumferential composite was superior to that of the GF circumferential composite. The glass transition temperature of the TBF circumferential composite was the highest, which indicated that the TBF circumferential composite had the best plasticizing properties and better elasticity. 

As a result, the interface between the bamboo fiber and resin can be improved by reducing the TBF diameter and increasing the specific surface area, making the composite possess high mechanical properties, which can provide the technology guidelines for the process and application of TBF.

## Figures and Tables

**Table 1:** Mechanical properties of composites made of different fibers.

Index	Density (g/cm^3^)	Tensile Strength (MPa)	Shearing Strength (MPa)	Bending Properties		
				Strength (MPa)	Modulus (MPa)	Surface Strain (%)
TBF-NOL *	0.974 (0.09)	45.62 (4.30)	18.31 (1.89)	105.41 (12.05)	3523.24 (328.01)	2.03 (0.21)
JF-NOL	1.088 (0.08)	128.53 (9.51)	22.31 (1.85)	114.33 (10.76)	2159.78 (157.36)	8.29 (0.68)
GF-NOL	1.901 (0.02)	885.81 (12.47)	37.53 (0.86)	828.17 (28.22)	12,508.46 (158.86)	11.79 (0.26)

* TBF-NOL: NOL composites made of twisting bamboo fiber; JF-NOL: NOL composites made of jute fiber; GF-NOL: NOL composites made of glass fiber. The standard deviations are listed in the parentheses.

**Table 2:** *k* values of NOL rings of different composites.

	*k* Value		
	40 °C	120 °C	200 °C
TBF-NOL	154.42 (17.16)	190.61 (21.04)	4.46 (0.44)
JF-NOL	354.89 (32.19)	2662.03 (240.06)	108.61 (8.53)
GF-NOL	252.33 (7.63)	827.01 (24.93)	14.75 (0.27)

## Data Availability

The data presented in this study are available on request from the corresponding author.

## References

[1] Joshi S.V., Drzal L.T., Mohanty A.K., Arora S. (2004). Are natural fiber composites environmentally superior to glass fiber reinforced composites?. Compos. Part A Appl. Sci. Manuf..

[2] Zelazinski T., Ekielski A., Tulska E., Vladut V., Durczak K. (2019). Wood dust application for improvement of selected properties of thermoplastic starch. INMATEH.

[3] Borowski P.F. (2021). Innovation strategy on the example of companies using bamboo. J. Innov. Entrep..

[4] Sun X., He M., Li Z. (2020). Novel engineered wood and bamboo composite for structural applications: State-of-art of manufacturing technology and mechanical performance evaluation. Constr. Build. Mater..

[5] Wang G., Shi S., Wang J.W., Yu Y., Cao S.P., Cheng H.T. (2011). Tensile properties of four types of individual cellulosic fibers. Wood Fiber Sci..

[6] Chen H., Yu Y., Zhong T., Wu Y., Li Y., Wu Z., Fei B. (2017). Effect of alkali treatment on microstructure and mechanical properties of individual bamboo fibers. Cellulose.

[7] Osorio L., Trujillo E., Lens F., Ivens J., Verpoest I., Van Vuure A. (2018). In-depth study of the microstructure of bamboo fibres and their relation to the mechanical properties. J. Reinf. Plast. Compos..

[8] Gujjala R., Ojha S., Acharya S., Pal S. (2014). Mechanical properties of woven jute–glass hybrid-reinforced epoxy composite. J. Compos. Mater..

[9] Li Y., Xie L., Ma H. (2015). Permeability and mechanical properties of plant fiber reinforced hybrid composites. Mater. Des..

[10] Zhang Z., Li Y., Chen C. (2017). Synergic effects of cellulose nanocrystals and alkali on the mechanical properties of sisal fibers and their bonding properties with epoxy. Compos. Part A Appl. Sci. Manuf..

[11] Wang C.C., Cheng H.T., Xian Y., Wang G., Zhang S.B. (2017). Improving dynamic mechanical property of bamboo pulp fiber reinforced epoxy resin composite treated by nano calcium carbonate. Trans. Chin. Soc. Agric. Eng..

[12] Qian S., Wang H., Zarei E., Sheng K. (2015). Effect of hydrothermal pretreatment on the properties of moso bamboo particles reinforced polyvinyl chloride composites. Compos. Part B Eng..

[13] Saba N., Jawaid M., Alothman O.Y., Paridah M., Hassan A. (2015). Recent advances in epoxy resin, natural fiber-reinforced epoxy composites and their applications. J. Reinf. Plast. Compos..

[14] Mittal V., Saini R., Sinha S. (2016). Natural fiber-mediated epoxy composites—A review. Compos. Part B Eng..

[15] Wang G., Cheng H.T., Gu S.H., Wang C.C., Zhang W.F. (2018). Classification and Application Status of Bamboo Profile. China For. Prod. Ind..

[16] Hebel D.E., Javadian A., Heisel F., Schlesier K., Griebel D., Wielopolski M. (2014). Process-controlled optimization of the tensile strength of bamboo fiber composites for structural applications. Compos. Part B Eng..

[17] Yu Z.X., Jiang Z.H., Wang G., Zhang W.F., Chen F.M. (2012). Mechanical properties of laminated bamboo scrimber in hygrothermal environment. J. Cent. South Univ. For. Technol..

[18] Kushwaha P.K., Kumar R. (2010). Studies on water absorption of bamboo-epoxy composites: Effect of silane treatment of mercerized bamboo. J. Appl. Polym. Sci..

[19] Xu J.Z., Qiao M., You B., Wang X.Y. (2009). The research of in-situ modeling process for fiber winding composite shell. Mater. Sci. Technol..

[20] Khennane A. (2013). Filament winding processes in the manufacture of advanced fibre-reinforced polymer (FRP) composites. Advanced Fibre-Reinforced Polymer (FRP) Composites for Structural Applications.

[21] Żelaziński T. (2021). Properties of biocomposites from rapeseed meal, fruit pomace and microcrystalline cellulose made by press pressing: Mechanical and physicochemical characteristics. Materials.

[22] Wang C., Smith L.M., Wang G., Shi S.Q., Cheng H., Zhang S. (2019). Characterization of interfacial interactions in bamboo pulp fiber/high-density polyethylene composites treated by nano CaCO_3_ impregnation modification using fractal theory and dynamic mechanical analysis. Ind. Crop. Prod..

